# Functional performance recovery after individualized nutrition therapy combined with a patient-tailored physical rehabilitation program versus standard physiotherapy in patients with long COVID: a pilot study

**DOI:** 10.1186/s40814-023-01392-1

**Published:** 2023-09-28

**Authors:** Stijn Roggeman, Berenice Gabriela Jimenez Garcia, Lynn Leemans, Joy Demol, Janne Geers, Ann De Smedt, Koen Putman, Marc Schiltz, David Beckwée, Elisabeth De Waele

**Affiliations:** 1 Department of Physical Medicine and Rehabilitation, Vrije Universiteit Brussel (VUB), Universitair Ziekenhuis Brussel (UZ Brussel), Laarbeeklaan, 101, 1090 Brussels, Belgium; 2 Department of Clinical Nutrition and Dietetics, Vrije Universiteit Brussel (VUB), Universitair Ziekenhuis Brussel (UZ Brussel), Laarbeeklaan, 101, 1090 Brussels, Belgium; 3 Research Group Rehabilitation Research (RERE), Vrije Universiteit Brussel, Laarbeeklaan, 103, 1090 Brussels, Belgium; 4 STIMULUS Consortium (reSearch and TeachIng neuroModULation Uz bruSsel), Vrije Universiteit Brussel (VUB), Laarbeeklaan, 103, 1090 Brussels, Belgium; 5 Interuniversity Centre for Health Economics Research (I-CHER), Vrije Universiteit Brussel (VUB), Laarbeeklaan, 103, 1090 Brussels, Belgium

**Keywords:** Long COVID, Physical exercise, Nutrition, Rehabilitation

## Abstract

**Background:**

Long COVID is suggested to be present in 14 to 43% of COVID 19-survivors. Literature on this new condition states a need for a multidisciplinary approach including physical exercise and nutrition. The aim of the current pilot study is to investigate the feasibility of the proposed protocol to prepare for a randomized controlled study that addresses the effectiveness of a personalized multimodal treatment compared to standard physiotherapy.

**Methods:**

This is a protocol of the UNLOCK (Nutrition and LOComotoric rehabilitation in long COVID) study, a pragmatic, single center, randomized controlled pilot trial with two groups. Patients with persisting symptoms related to a SARS-CoV-2 infection will receive either standard physiotherapy or a personalized multimodal treatment for a period of 12 weeks, consisting of individualized physical exercise program combined with individualized nutritional therapy. They will be followed-up at 6, 12, and 18 weeks after randomization.

**Discussion:**

A multidisciplinary approach for dealing with long COVID is needed. Because of the lack of clear data and the fact that this is a very heterogenic group, we aim to prepare and optimize a randomized controlled study that addresses the effectiveness of a personalized multimodal treatment.

**Trial registration:**

ClinicalTrials.gov Identifier: NCT05254301 (since February 24, 2022).

## Background

The rapid spread of SARS-CoV-2 virus has already led to more than 767 million confirmed cases including over 6.9 million deaths worldwide as of 28 June 2023 [1]. The acute phase of infection resulted in variable clinical presentations, from asymptomatic to severe illness requiring admission at an intensive care unit. Now we are facing the challenge of long COVID (aka post COVID-19 syndrome). According to the World Health Organization (WHO), this condition occurs in individuals with a history of probable or confirmed SARS CoV-2 infection, usually 3 months from the onset of COVID-19 with symptoms and that last for at least 2 months and cannot be explained by an alternative diagnosis [2]. Recent meta-analysis suggests a global prevalence of 43% with higher risk if the patient was hospitalized compared to non-hospitalized, respectively 54% and 34% [3]. The most common complaints are fatigue, headache, attention disorder (‘brain fog’), hair loss and dyspnea. Otherwise, exertional intolerance/post-exertional malaise is frequently seen next to other persistent symptoms [3, 4]. Altogether, these complaints have an impact on the physical, cognitive and mental functioning and lead to reduced autonomy and quality of life [5]. One research group showed that 2 years post infection, the burden of symptomatic sequelae remained high, and COVID-survivors had lower health status than the general population [6]. How to deal with this new health condition is still a matter of debate and research on the treatment options are ongoing. Many interventions are based on research performed in patients presenting similar difficulties but caused by other diseases (e.g., COPD, cancer rehabilitation, chronic fatigue syndrome…) [7, 8]. In this regard, the most recent WHO guideline on the clinical management of COVID-19 recognizes that this multisystem disease may require multidisciplinary rehabilitation [9]. Besides the ‘physical’ problems, most patients show some degree of weight loss, muscle wasting, and malnutrition. Reduced food intake, loss of appetite, COVID-19-related loss of taste and smell, fever, inflammation, higher catabolism, endocrine dysfunction, and immobilization can all cause weight loss and associated malnutrition during the acute phase of the disease. Additionally, small percentages of long COVID patients suffer from gastro-intestinal symptoms such as nausea, vomiting, diarrhea, or other symptoms that limit their food intake, such as loss of smell and taste, even 2 years after the SARS-CoV-2 infection [10]. Three studies showed a frequency between 29 and 52% of weight loss of ≥ 5% during hospitalisation, which together with impaired functional status and inflammation is indicative of cachexia [11–14]. A systematic review and meta-analysis of 5407 COVID-19 patients showed a pooled prevalence of 48% for sarcopenia, characterized by low skeletal muscle mass and reduced strength. Another systematic review and meta-analysis studied the prevalence of body composition abnormalities in 30 studies and found a prevalence of high visceral adipose tissue (VAT) of 16.1%, 75% of COVID-19 patients had high subcutaneous adipose tissue (SAT) and 31.5% to 50.3% had a high VAT:SAT ratio [15]. When Bio-electrical Impedance Analysis (BIA) was performed, 50.9% of patients showed a high fat mass percentage [16].

Consequently, the combination of low muscle mass and excess adipose tissue should be targeted as a treatment point of engagement. Indeed, nutritional interventions to counter cachexia in patients with different medical conditions vary and require specific attention points and targets. As proven by the plethora of guidelines from the European Society of Clinical Nutrition and Metabolism (ESPEN), guidance of patients is key to achieve well-defined endpoints, but multiple clinical challenges exist in different disease types [17]. Moreover, as shown in previous work, the combination of physical therapy and nutrition is well known in the treatment of low muscle mass in cancer [18].

Our hypothesis is that an individualized nutritional therapy, combined with individualized physical rehabilitation program may lead to a faster improvement of functional performance compared to a standard physiotherapy program in patients suffering from long-term effects of COVID-19. Before running a large randomized controlled trial, it seems necessary to perform a pilot study that assesses the feasibility of the proposed intervention and to recruit patients for such trial. Main focus in term of feasibility will be the study burden on the participant and the recruitment rates. Second, the gathered results could help us in understanding how long COVID impacts daily life and the ability to recover through multidisciplinary personalised treatment. This data would allow us to better calculate the effect sizes required for sample size estimation. The findings of this pilot study will help us decide about proceeding to a future definitive randomized controlled trial (RCT).

## Methods/design

### Aim and trial design

The aim with the present protocol is to prepare a larger-scaled RCT that addresses the effectiveness of a personalized multimodal treatment (PMT). This pilot will offer us necessary information on the feasibility of the future experiment.

The UNLOCK trial will investigate whether a PMT, consisting of individualized physiotherapy and nutritional therapy, is able to improve functional performance recovery faster than standard physiotherapy alone. This trial is a pragmatic, single-center, randomized controlled pilot study with 2 parallel groups: standard physiotherapy and the PMT. The trial will be performed at Universitair Ziekenhuis Brussel, Brussels, Belgium, and conducted in accordance with the Declaration of Helsinki.

The protocol conforms to the Standard Protocol Items: Recommendations for Interventional Trials (SPIRIT) guidelines.

### Patient involvement and ethical considerations

The protocol with all proposed assessments is discussed with our Patient Advisory Board. It is also approved by the Medical Ethics Committee of UZ Brussel/Vrije Universiteit Brussel (BUN: 1432022000068).

### Eligibility criteria and recruitment strategy

We search adult patients experiencing persistent symptoms > 12 weeks after initial SARS-CoV-2 infection.

 This criterion is in line with the definition of long COVID by the WHO [2]. Since we are dealing with a very heterogenic group, we have limited our eligibility criteria to the minimum to include as many long COVID patients as possible (Table 1).

**Table 1:** Eligibility criteria

Inclusion criteria	Exclusion criteria
Able to understand and sign written informed consent in Dutch, French, or English	Patient with medical history of any other disease besides long COVID that could explain the symptoms
Adult (≥ 18 years old)	Patient is unable to undergo a rehabilitation program due to comorbidities (e.g., major cardiovascular disease such as myocarditis or severe dementia), as decided on by the medical study team members
Laboratory (PCR and/or serology) confirmed infection with SARS-CoV-2 or probable diagnosis based on the clinical diagnosis	Patient currently benefiting from physiotherapy sessions with focus on motor and/or respiratory (independent for underlying condition)
Persisting functional difficulties and symptoms: exercise intolerance and/or fatigue and/or muscle pain beyond 12 weeks beside other COVID-related symptoms (e.g., loss of taste and/or smell)	Patients with metabolic disorders such as inborn errors of metabolism or badly controlled metabolic disorders (e.g., diabetes mellitus), or severe gastro-intestinal conditions (e.g., short bowel syndrome)
Patient enrolled in a Belgian health insurance	Patients unable to comprehend oral and/or written instructions, questionnaires in English, French, or Dutch

Participants will be recruited through referral from health care workers, spreading of leaflets throughout the campus and online and through members of the advisory boards. All interested individuals can contact the research team for further information. After this initial contact, a formal clinical screening check will be performed. During this, the medical practitioner will perform a standard anamnesis about their COVID-infection and persisting symptoms, medical history, use of medication and perform a standard clinical examination. He will check the eligibility of the possible participant conform the in- and exclusion criteria and if necessary, refer to a specialist for advice and/or further investigation (e.g., in the presence of cardiac without available evaluation). Also, patients who fail to meet the Get Active Questionnaire (GAQ) [19] will not be considered eligible. If the patient is eligible, the medical doctor will obtain informed consent.

### Primary feasibility outcomes

The first aim of this pilot study is to test trial procedures and processes. This implies running through the full procedures, with the patients, to detect possible challenges. Our main focus in terms of feasibility are patient study burden and recruitment and attrition rates. The patients will be asked during the last visit if the study procedures are acceptable, which will help to make adaptation of the protocol. Next, we should be able to observe recruitment rates and intervene, try to persuade and discuss reasons with the patients to avoid low responses. In that respect, recruitment fewer than anticipated (10/month) and an attrition of 20% will automatically trigger a discussion with the steering committee on how to counter these inappropriate rates. A second aim is to get a better understanding of the data, which is necessary because of the lack of data in this population.

### Assessment battery of the main trial

**Fig. 1:**
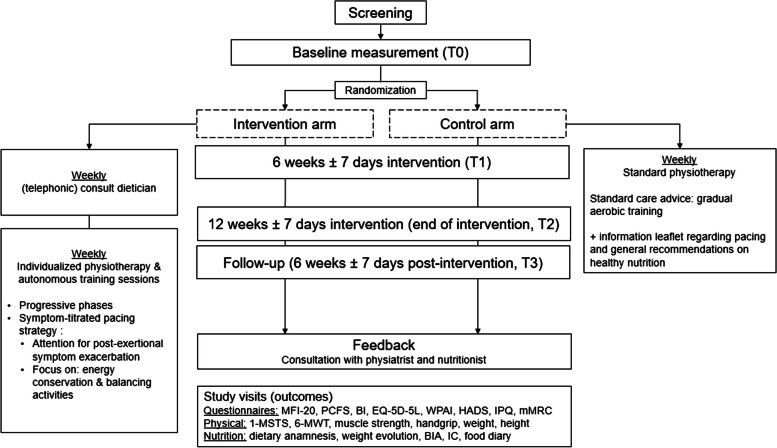
Trial flow chart

All parameters will be measured in both intervention and control group at baseline (T0), week 6 (T1) and 12 (T2) of intervention and at 6 weeks follow-up (T3) (Fig. 1).

#### Primary outcome measure

Improvement of physical performance will be assessed using the *1-min sit-to-stand (1-MSTS)* test together with additional secondary and exploratory outcome measures. The 1-MSTS test is indicative of functional performance and has proven valid and reliable for several medical conditions, but most studies are done in patients with COPD [20, 21].

#### Secondary outcome measures

Based on literature and discussions with our Patient Advisory Board, it seems that fatigue is the most frequently experienced problem. For this reason, the evaluation of fatigue will be our most important secondary outcome. This will be done using the *Multi-dimensional Fatigue Inventory (MFI-20)*. This questionnaire covers the following dimensions: general fatigue, physical fatigue, reduced activity, reduced motivation and mental fatigue. It is also validated in several languages and for different populations, including cancer, post-ICU or Parkinson’s disease [22–25].

Next, health-related quality of life will be measured by the *5-level EuroQol 5 dimensions (EQ-5D-5L)* which comprises 5 dimensions: mobility, self-care, usual activities, pain/discomfort, and anxiety/depression. This test has proven good feasibility, reliability and validity [26].

Implications on relevant aspects of daily life after COVID infection will be evaluated for changes using the *Post-COVID-19 Functional Status Scale (PCFS)*, which is specifically developed for COVID patients by Klok and colleagues [27]. Work capability will be assessed using the *Work Productivity and Activity Impairment (WPAI)* questionnaire. This consists of 6 questions asking about the decline in productivity and activity in the past 7 days. The WPAI is considered the best-validated questionnaire for determining health-related work productivity and has been validated in various diseases (e.g., allergic rhinitis, gastro-esophageal reflux disease, chronic hand dermatitis, ankylosing spondylitis) [28, 29].

Lastly, mental status will be measured using the *Hospital Anxiety and Depression Scale (HADS),* which assesses both anxiety and depression, each with 7 questions. This questionnaire is widely used for many different conditions, including COVID, and is validated in multiple languages [30, 31].

#### Explanatory measures

Explanatory measures would be sex, adherence, illness perceptions questionnaire (IPQ) and duration of symptoms. During intake, assessors will also retrieve descriptive variables such as COVID-19 hospitalization, length of hospitalization, ICU stay and length, and duration of sickness leave. Furthermore, the following explanatory outcomes will be measured during assessments:

Changes in *physical performance* will be assessed by means of the 6-min walk test (6-MWT) and muscle strength. These measurements will help confirm the results of the 1-MSTS test. The 6-MWT is used to evaluate an individual's gait pattern, speed and overall endurance and initially designed for patients with cardiopulmonary issues [25]. This test has proven excellent test-retest reliability in diseases like geriatrics, osteoarthrosis and stroke [32–35]. Muscle strength will be measured both by handgrip strength using a Martin Vigorimeter and the general muscle strength (shoulder abduction, elbow flexion, wrist extension, hip flexion, knee extension, ankle dorsiflexion bilateraly) using the Handheld dynamometer (MicroFet).

*Dyspnea* will be evaluated by the modified Medical Research Council (mMRC) dyspnea questionnaire. The questionnaire was initially invented and validated on patients with COPD and is based on the GOLD-criteria [36].

The Barthel Index will evaluate to which extent a person needs help performing *activities of daily living*. This parameter can possibly further explain results of the EQ-5D-5L and PCFS questionnaires.

*Baseline nutritional status and changes in nutritional parameters* will be measured. These parameters are important because they provide a look inside the patient and can help to explain fatigue, lack of energy or return of energy. They include

- ◦ Adequacy of feeding: this will be calculated for energy, proteins, fatty acids, carbohydrates, and fiber. It is defined as the average intake divided by the individual nutritional need. This is described in detail in *Task 1. Individualized nutritional therapy.*
- ◦ Phase angle: bioimpedance analysis is performed by a phase sensitive device (BIA 101 BIVA® PRO AKERN srl, Florence, Italy) working with alternating sinusoidal electric current of 245 µA at a frequency of 50 kHz. For the measurement, participants are supine with limbs slightly spread apart from the body. Very low intrinsic impedance (< 30 Ω) disposable electrodes (Biatrodes™ Akern Srl; Florence, Italy) are placed on the right side at metacarpal and metatarsal sites of the right wrist and ankle [37]. The phase angle (PhA) correlated with better survival in cancer patients and critically ill patients and is shown to increase with resistance training in older adults. It is a measure of cell and thereby body vitality [38–40].
- ◦ Fat-to-fat free mass ratio: this parameter compares fat mass to fat-free mass which includes bone muscle mass.

These variables will be used to check for responders/non-responders or define subgroups and their possible correlation to primary and secondary endpoints.

### Intervention

The study compares 2 treatment options for patients with long COVID: standard care consisting of 18 physiotherapy sessions with a physiotherapist of choice and the personalized multimodal treatment (PMT). The PMT will consist of a combination of individualized nutritional therapy (task 1) and a physical training program (task 2). The PMT considers evidence- and practice-based elements such as individual symptom-contingent pacing, self-management, and home-based functional training.

### Task 1: individualized nutritional therapy

A nutritional interview (45 min) will be performed at baseline. During this interview, a nutritional anamnesis is performed based on standard dietetic practice to assess the usual intake and usual eating patterns. Additionally, the BMI and weight evolution since the COVID-19 infection and/or in the last 3–6 months are assessed. If during this anamnesis, any exclusion criteria are discovered or presumed (e.g., severe gastro-intestinal conditions that lead to rapid weight loss or absorption issues), the participation will be ended or postponed until the medical team can decide if it is safe for the participant. All participants will complete a food diary (last 3 days), that will be analyzed by registered dietitians using the Belgian Food Composition Database Nubel PRO. The control group will be given general nutritional advice in the form of a leaflet with information about healthy nutrition. The intervention group will be followed-up by one of the study dieticians during a weekly tele-consultation in the first 12 weeks (between T0 and T2), aiming at better guidance of participants throughout the intervention period. This is a low-threshold intervention with proven benefit and feasibility, validated in our former trial [18].

The focus of the individual nutritional therapy will be to match energy and protein intake with the individualized needs, to support the physical rehabilitation and the maintenance of increase of muscle mass. Additionally, the dietitians will focus on the nutritional guidelines from the Belgian High Health Council to ensure a healthy, evidence-based approach. The caloric target is defined by the Resting Energy Expenditure (REE) in kcal/day measured with indirect calorimetry (Q-NRG™ Metabolic Monitor, COSMED) multiplied by a physical activity level (PAL) [41]. The Q-NRG showed prolonged hypermetabolism in COVID-19 ICU patients up to 7 weeks, showing a need to accurately measure the REE [42]. For protein, 0.83 g/kg bodyweight is used, unless a higher protein need is recommended due to specific pathology or in case of sarcopenia. Carbohydrate, fatty acids and fiber needs are defined as 50–55% of the energy requirements (En%), 30–35 En% and 3 En% respectively. The use of adequacy of feeding based on indirect calorimetry, also referred to as the Tight Caloric Control Concept, is a well-known concept in the world of Intensive Care where it proved a survival benefit in a recent meta-analysis [43]. This concept was translated by our Nutritional Research Team in a prospective randomized control trial to battle cancer-related cachexia. It proved a significant benefit [18].

If the participant requires any other nutritional interventions, e.g., in case of food intolerances or other conditions, the dietitians will develop individual nutritional targets. If there is an indication for weight-loss it is important that the interventions do not interfere with the treatment of the long COVID symptoms (e.g., adequate energy intake to combat fatigue versus energy restricted diet for weight-loss). In cases like this, the dietitians and the participant will discuss priorities and adjust the intervention accordingly. As this is a pragmatic trial, the dietitians will use any evidence-based tools that they would use in usual, individualized care. The individualized nutritional therapy can consist of food, oral supplements, enteral nutrition or in very rare cases parenteral nutrition.

### Task 2: Patient-tailored physical exercise program

The exercise program implements the recommendations of the World Physiotherapy Organization [44]. The participants will train a maximum of two to three times a week for 12 weeks. A trained physiotherapist will supervise 18 sessions. His goal will be teaching the patients to independently perform the program as well as increasing the training load based on individual assessments and symptoms. The program consists of different progressive phases, including preparation for return to exercise (breathing and stretching), low-intensity activity, moderate-intensity analytical and functional exercises and return to pre-COVID activity regime. A symptom-titrated pacing strategy will be implemented to account for post-exertional symptom exacerbation. As recommended by World Physiotherapy [44], people with post-exertional symptom exacerbation (PESE) will be identified by asking about their symptoms and the impact of physical, cognitive and social activities on symptoms 12 h or longer after exertion. A 5-item questionnaire, i.e., a subscale of the De Paul Symptom Questionnaire will be used to assess PESE. These five questions are also recommended by the National Institutes of Health/Centers for Disease Control and Prevention Common Data Elements post-exertional malaise working group [44]. A score of 2 on both frequency and severity on any items 1 to 5, is indicative of post-exertional malaise [45]. Five supplemental questions will also be used to examine duration, recovery, and exercise exacerbation. If symptom exacerbation is present, symptom stabilization is of prime importance. Thus, symptom-contingent pacing [46] will be used to engage in activities guided by perceived symptom levels to avoid worsening symptoms, conserve energy, and enable participation in meaningful activities. Therefore, focus will be put on energy conservation and balancing activities with rest to avoid further exacerbation of symptoms (phase 1: breathing and stretching exercises). Subsequently, moving to next stages in the exercise program will be based on measures of perceived exertion and a visual analogue scale for symptoms. This is in line with the current insights, reported by the World Physiotherapy briefing paper 9 [44]. During the program, the professionals involved with patients in the intervention group will be equipped with a documentation map with all the information, materials, formats, and exercises, including quick sheets with do’s and don’ts during training, motivation cards for goalsetting and evaluation, log with evaluative and motivational questions for the participant regarding the intensity, feasibility, pleasantness and training progress of the trainings/program. The focus will be on personal goals and achievements by means of the training program and coping strategies when adherence or motivation is low.

The participants of the control group will be given a prescription for 18 sessions of physiotherapy with standard care advice: gradual aerobic training. In addition, they will receive an information leaflet with information regarding symptom-contingent pacing and general recommendations on healthy nutrition. Participants are also asked to keep track of their training sessions through an exercise and food diary and if they did dietary modifications.

### Trial randomization and blinding

Once a patient signs the informed consent and after the first study visit, he/she will be randomized into one of both treatment arms by one of the investigators using an interactive web response system (IWRS). Patients will be randomized in a 1:1 ratio, using permuted block randomization with blocks of variable sizes (2–4-6) into one of the two intervention parallel groups.

The statistician will be blinded to group allocation. Outcome assessors will be blinded to the maximal extent possible. Participants will of course be aware of the intervention received. Regarding this, participants will be asked not to communicate with the assessors about the intervention received. The therapists providing the experimental intervention will not be involved in the usual care treatment, and vice versa.

### Sample size

For this pilot we aim for 52 participants. This is in line with Whitehead et al. who recommends a sample size of 25 per treatment arm of interest in a pilot study, accounting for a standardized mean difference of 0.2 in the future definitive RCT with a power of 90%, and two-sided 5% significance [47]. Considering an attrition rate of 20%, 66 patients will be randomized.

### Statistical analysis

The focus is primarily qualitative and unstructured but there is a minor but important statistical focus which is descriptive, which focuses on the full distribution of observations in general and the dispersion in particular. All parameters will be analyzed descriptively by treatment arm, there will be no formal hypothesis testing and a repeated measures model will be used to derive an estimate of the treatment effect. While the actual analysis can be performed as planned, because all data required for that should be available, any statistical test would presumably be underpowered.

### Data handling and monitoring

To define data management practices the data manager developed a clinical data management plan (CDMP), that was reviewed by the principal investigator and the trial monitor. Besides the CDMP, multiple standard-operating procedures were developed for screening, randomization, assessments during study visits, and individual nutritional therapy. In case of protocol deviations, these will be reported in the Trial Master File and the ethics committee will be notified.

## Discussion

There is need for a multidisciplinary approach when dealing with long COVID (post-COVID syndrome) manifestations. The UNLOCK study will investigate whether a personalized multimodal treatment (tailored physiotherapy and individual nutritional therapy) is able to improve functional performance recovery faster than standard physiotherapy alone. The choice for functional endpoints is motivated by the fact that patients suffering long COVID experience difficulties performing their daily activities whether because of exertional intolerance (fatigue, dyspnea, widespread pain, deconditioned state…) or cognitive difficulties (attention and memory deficits, brain fog…).

The rationale for the combination of both interventions (physical therapy and nutritional optimization) is based on our clinical expertise and the feedback from our long COVID patients regarding the need for a multidisciplinary approach to account for their complaints (including feelings of fatigue, post-exercise symptom exacerbation, and loss of smell and taste). In addition, the rationale for not including a separate study arm (only personalized physical exercise program), which would allow us to investigate the contribution of the nutrition component, is based on feasibility issues as well as to respond to the cry-out and sense of urgency, reported by the long COVID patient community.

Because of the lack of clear data on long COVID patients and the fact that this group is very heterogenic, we aim with the present protocol to prepare a randomized controlled study that addresses the effectiveness of a personalized multimodal treatment. This pilot will offer us necessary information on the feasibility of the future experiment.

## Data Availability

Not applicable in this section.

## Abbreviations

1-MSTS: 1-Minute sit-to-stand test
6-MWT: 6-Minute walk test
AEs: Adverse events
CDMP: Clinical data management plan
eCRF: Electronic case report form
En%: Energy requirements
ESPEN: European Society of Clinical Nutrition and Metabolism
EQ-5D-5L: 5-Level EuroQol 5 dimensions
HADS: Hospital Anxiety and Depression Scale
GAQ: Get Active Questionnaire
IPQ: Illness perceptions questionnaire
IWRS: Interactive web response system
MFI-20: Multi-dimensional Fatigue Inventory
mMRC: Modified Medical Research Council
PCFS: Post-COVID-19 Functional Status
PESE: Post-exertional symptom exacerbation
PMR: Physical Medicine and Rehabilitation
PMT: Personalized multimodal treatment
RCT: Randomized controlled trial
SAEs: Severe adverse events
WPAI: Work Productivity and Activity Impairment

## References

[1] WHO COVID-19 Dashboard. Geneva: World Health Organization. https://covid19.who.int. Accessed 28 Jun 2023

[2] World Health Organization. A clinical definition of post COVID-19 condition by a Delphi consensus OAJ. https://www.who.int/news-room/questions-and-answers/item/coronavirus-disease-(covid-19)-post-covid-19-condition. Accessed 10 Sept 2022.

[3] Chen C, Haupert SR, Zimmermann L, Shi X, Fritsche LG, Mukherjee B. Global Prevalence of Post-Coronavirus Disease 2019 (COVID-19) Condition or Long COVID: A Meta-Analysis and Systematic Review. J Infect Dis. 2022;226(9):1593–1607. 10.1093/infdis/jiac136 PMC9047189 35429399

[4] Lopez-Leon S, Wegman-Ostrosky T, Perelman C, Sepulveda R, Rebolledo PA, Cuapio A (2021). More than 50 long-term effects of COVID-19: a systematic review and meta-analysis. Sci Rep.

[5] Walle-Hansen MM, Ranhoff AH, Mellingsæter M, Wang-Hansen MS, Myrstad M (2021). Health-related quality of life, functional decline, and long-term mortality in older patients following hospitalisation due to COVID-19. BMC Geriatr.

[6] Huang L, Li X, Gu X, Zhang H, Ren L, Guo L (2022). Health outcomes in people 2 years after surviving hospitalisation with COVID-19: a longitudinal cohort study. Lancet Respir Med.

[7] Vaes AW, Goërtz YMJ, Van Herck M, Machado FVC, Meys R, Delbressine JM, et al. Recovery from COVID-19: a sprint or marathon? 6-month follow-up data from online long COVID-19 support group members. ERJ Open Res. 2021;7(2):00141–2021. 10.1183/23120541.00141-2021 PMC8012818 34041295

[8] Gutenbrunner C, Nugraha B, Martin LT (2021). Phase-adapted rehabilitation for acute coronavirus disease-19 patients and patient with long-term sequelae of coronavirus disease-19. Am J Phys Med Rehabil.

[9] World Health Organization. Living guidance for clinical management of COVID-19. https://www.who.int/publications/i/item/WHO-2019-nCoV-clinical-2021-2. Accessed 10 Sept 2022. 35917394

[10] Rahmati M, Udeh R, Yon DK, Lee SW, Dolja-Gore X, Mc EM (2023). A systematic review and meta-analysis of long-term sequelae of COVID-19 2-year after SARS-CoV-2 infection: A call to action for neurological, physical, and psychological sciences. J Med Virol.

[11] Pironi L, Sasdelli AS, Ravaioli F, Baracco B, Battaiola C, Bocedi G (2021). Malnutrition and nutritional therapy in patients with SARS-CoV-2 disease. Clin Nutr.

[12] Di Filippo L, De Lorenzo R, D'Amico M, Sofia V, Roveri L, Mele R (2021). COVID-19 is associated with clinically significant weight loss and risk of malnutrition, independent of hospitalisation: a post-hoc analysis of a prospective cohort study. Clin Nutr.

[13] Allard L, Ouedraogo E, Molleville J, Bihan H, Giroux-Leprieur B, Sutton A, et al. Malnutrition: percentage and association with prognosis in patients hospitalized for coronavirus disease 2019. Nutrients. 2020;12(12):3679. 10.3390/nu12123679 PMC7761464 33260603

[14] Evans WJ, Morley JE, Argilés J, Bales C, Baracos V, Guttridge D (2008). Cachexia: a new definition. Clin Nutr.

[15] Montes-Ibarra M, Orsso CE, Limon-Miro AT, Gonzalez MC, Marzetti E, Landi F (2023). Prevalence and clinical implications of abnormal body composition phenotypes in patients with COVID-19: a systematic review. Am J Clin Nutr.

[16] Stevanovic D, Zdravkovic V, Poskurica M, Petrovic M, Cekerevac I, Zdravkovic N (2022). The role of bioelectrical impedance analysis in predicting COVID-19 outcome. Front Nutr.

[17] Singer P, Blaser AR, Berger MM, Alhazzani W, Calder PC, Casaer MP (2019). ESPEN guideline on clinical nutrition in the intensive care unit. Clin Nutr.

[18] De Waele E, Mattens S, Honoré PM, Spapen H, De Grève J, Pen JJ. Nutrition therapy in cachectic cancer patients. The Tight Caloric Control (TiCaCo) pilot trial. Appetite. 2015;91:298–301. 10.1016/j.appet.2015.04.049 25912786

[19] Petrella AFM, Gill DP, Petrella RJ (2018). Evaluation of the Get Active Questionnaire in community-dwelling older adults. Appl Physiol Nutr Metab.

[20] Bohannon RW, Crouch R (2019). 1-Minute Sit-to-Stand Test: Systematic review of procedures, performance, and clinimetric properties. J Cardiopulm Rehabil Prev.

[21] Vaidya T, de Bisschop C, Beaumont M, Ouksel H, Jean V, Dessables F (2016). Is the 1-minute sit-to-stand test a good tool for the evaluation of the impact of pulmonary rehabilitation? Determination of the minimal important difference in COPD. Int J Chron Obstruct Pulmon Dis.

[22] Smets EM, Garssen B, Bonke B, De Haes JC (1995). The Multidimensional Fatigue Inventory (MFI) psychometric qualities of an instrument to assess fatigue. J Psychosom Res.

[23] Gentile S, Delarozière JC, Favre F, Sambuc R, San Marco JL (2003). Validation of the French 'multidimensional fatigue inventory' (MFI 20). Eur J Cancer Care (Engl).

[24] Wintermann GB, Rosendahl J, Weidner K, Strauß B, Hinz A, Petrowski K (2018). Fatigue in chronically critically ill patients following intensive care - reliability and validity of the multidimensional fatigue inventory (MFI-20). Health Qual Life Outcomes.

[25] Elbers RG, van Wegen EE, Verhoef J, Kwakkel G (2012). Reliability and structural validity of the Multidimensional Fatigue Inventory (MFI) in patients with idiopathic Parkinson's disease. Parkinsonism Relat Disord.

[26] Xu RH, Keetharuth AD, Wang LL, Cheung AW, Wong EL (2022). Measuring health-related quality of life and well-being: a head-to-head psychometric comparison of the EQ-5D-5L, ReQoL-UI and ICECAP-A. Eur J Health Econ.

[27] Klok FA, Boon G, Barco S, Endres M, Geelhoed JJM, Knauss S, et al. The Post-COVID-19 Functional Status scale: a tool to measure functional status over time after COVID-19. Eur Respir J. 2020;56(1):2001494. 10.1183/13993003.01494-2020 PMC7236834 32398306

[28] Prasad M, Wahlqvist P, Shikiar R, Shih YC (2004). A review of self-report instruments measuring health-related work productivity: a patient-reported outcomes perspective. Pharmacoeconomics.

[29] Reilly MC, Gooch KL, Wong RL, Kupper H, van der Heijde D (2010). Validity, reliability and responsiveness of the Work Productivity and Activity Impairment Questionnaire in ankylosing spondylitis. Rheumatology (Oxford).

[30] Nikolovski A, Gamgoum L, Deol A, Quilichini S, Kazemir E, Rhodenizer J, Oliveira A, Brooks D, Alsubheen S. Psychometric properties of the Hospital Anxiety and Depression Scale (HADS) in individuals with stable chronic obstructive pulmonary disease (COPD): a systematic review. Disabil Rehabil. 2023:1-9. 10.1080/09638288.2023.2182918 36861817

[31] Fernández-de-Las-Peñas C, Rodríguez-Jiménez J, Palacios-Ceña M, de-la-Llave-Rincón AI, Fuensalida-Novo S, Florencio LL, et al. Psychometric Properties of the Hospital Anxiety and Depression Scale (HADS) in previously hospitalized COVID-19 patients. Int J Environ Res Public Health. 2022;19(15):9273. 10.3390/ijerph19159273 PMC9367824 35954630

[32] ATS Committee on Proficiency Standards for Clinical Pulmonary Function Laboratories. ATS statement: guidelines for the six-minute walk test. Am J Respir Crit Care Med. 2002;166(1):111–7. 10.1164/ajrccm.166.1.at1102 12091180

[33] Steffen TM, Hacker TA, Mollinger L (2002). Age- and gender-related test performance in community-dwelling elderly people: Six-Minute Walk Test, Berg Balance Scale, Timed Up & Go Test, and gait speeds. Phys Ther.

[34] Kennedy DM, Stratford PW, Wessel J, Gollish JD, Penney D (2005). Assessing stability and change of four performance measures: a longitudinal study evaluating outcome following total hip and knee arthroplasty. BMC Musculoskelet Disord.

[35] Fulk GD, Echternach JL, Nof L, O'Sullivan S (2008). Clinometric properties of the six-minute walk test in individuals undergoing rehabilitation poststroke. Physiother Theory Pract.

[36] Wedzicha JA, Bestall JC, Garrod R, Garnham R, Paul EA, Jones PW (1998). Randomized controlled trial of pulmonary rehabilitation in severe chronic obstructive pulmonary disease patients, stratified with the MRC dyspnoea scale. Eur Respir J.

[37] Lukaski HC, Bolonchuk WW, Hall CB, Siders WA. Validation of tetrapolar bioelectrical impedance method to assess human body composition. J Appl Physiol (1985). 1986;60(4):1327–32. 10.1152/jappl.1986.60.4.1327 3700310

[38] Sandini M, Paiella S, Cereda M, Angrisani M, Capretti G, Famularo S (2023). Independent effect of fat-to-muscle mass ratio at bioimpedance analysis on long-term survival in patients receiving surgery for pancreatic cancer. Front Nutr.

[39] Lima J, Eckert I, Gonzalez MC, Silva FM (2022). Prognostic value of phase angle and bioelectrical impedance vector in critically ill patients: a systematic review and meta-analysis of observational studies. Clin Nutr.

[40] Campa F, Colognesi LA, Moro T, Paoli A, Casolo A, Santos L (2023). Effect of resistance training on bioelectrical phase angle in older adults: a systematic review with Meta-analysis of randomized controlled trials. Rev Endocr Metab Disord.

[41] Oshima T, Berger MM, De Waele E, Guttormsen AB, Heidegger CP, Hiesmayr M, et al. Indirect calorimetry in nutritional therapy. A position paper by the ICALIC study group. Clin Nutr. 2017;36(3):651–62. 10.1016/j.clnu.2016.06.010 27373497

[42] Niederer LE, Miller H, Haines KL, Molinger J, Whittle J, MacLeod DB (2021). Prolonged progressive hypermetabolism during COVID-19 hospitalization undetected by common predictive energy equations. Clin Nutr ESPEN.

[43] Duan JY, Zheng WH, Zhou H, Xu Y, Huang HB (2021). Energy delivery guided by indirect calorimetry in critically ill patients: a systematic review and meta-analysis. Crit Care.

[44] World Physiotherapy. World Physiotherapy Response to COVID-19 Briefing Paper 9. Safe rehabilitation approaches for people living with Long COVID: physical activity and exercise. London: World Physiotherapy; 2021.

[45] Borg GA (1982). Psychophysical bases of perceived exertion. Med Sci Sports Exerc.

[46] Nijs J, Paul L, Wallman K (2008). Chronic fatigue syndrome: an approach combining self-management with graded exercise to avoid exacerbations. J Rehabil Med.

[47] Whitehead AL, Julious SA, Cooper CL, Campbell MJ (2016). Estimating the sample size for a pilot randomised trial to minimise the overall trial sample size for the external pilot and main trial for a continuous outcome variable. Stat Methods Med Res.

